# Supplementary material for: Patients with coronary heart disease, dilated cardiomyopathy and idiopathic ventricular tachycardia share overlapping patterns of pathogenic variation in cardiac risk genes
Source: PeerJ. 2021 Jan 19;9:e10711. doi: 10.7717/peerj.10711 (PMC7821765; doi:10.7717/peerj.10711)
Supplement: Supplemental Information 1 [file peerj-09-10711-s001.docx]

**Supplemental File 1:**

**Criteria of diagnosis of patients. Inclusion/exclusion criteria**

Ventricular tachycardia (VT) refers to any rhythm faster than 100 (or 120) beats/min arising distal to the bundle of His.

Patients without clinical characteristics for D/HCM, CHD and other heart diseases but with at least one ECG-documented episode of VT were included in the idiopathic VT study group.

Exclusion criteria in this study meet patients with VT who had: electrolyte deficiencies (eg, [hypokalemia](http://emedicine.medscape.com/article/767448-overview), [hypocalcemia](http://emedicine.medscape.com/article/767260-overview), and [hypomagnesemia](http://emedicine.medscape.com/article/767546-overview)); systemic diseases that affect the myocardium (eg, sarcoidosis, myloidosis, [systemic lupus erythematosus](http://emedicine.medscape.com/article/809378-overview), hemochromatosis, and [rheumatoid arthritis](http://emedicine.medscape.com/article/331715-overview)); sympathomimetic agents, including IV inotropes and illicit drugs such as methamphetamine or cocaine; [Digitalis toxicity](http://emedicine.medscape.com/article/814404-overview), which can lead to biventricular tachycardia; drugs that prolong the QT interval (eg, class IA and class III antiarrhythmics, azithromycin, levofloxacin, and many others); these may cause [torsades de pointes](http://emedicine.medscape.com/article/1950863-overview); drugs that slow conduction velocity, particularly when underlying myocardial scar is present (eg, halothane and class IA and IC antiarrhythmics).

Clinical diagnosis of DCM was diagnosed according to the WHO/International Society and Federation of Cardiology Task Force clinical criteria as published by Richardson P, McKenna W, Bristow M, Maisch B, Mautner B, O’Connell J, Olsen E, Thiene G, Goodwin J, Gyarfas I, Martin I, Nordet P. Report of the 1995 World Health Organization/International Society and Federation of Cardiology Task Force on the Deﬁnition and Classiﬁcation of cardiomyopathies. Circulation 1996; 93:841–842.

DCM was defined as a myocardial disorder, characterized by systolic impairment and the left ventricular dilatation, in the absence of abnormal loading conditions (e.g. valve diseases, hypertension >160/100mm HG) or coronary artery disease (CAD; e.g. stenosis of > 50% in one or more major arteries). Further exclusion criteria were chronic alcohol abuse (more than 40 g/day for women and more than 80 g/day for men for more than 5 years) or other systemic diseases, diseases of the pericardium, congenital heart disease or pulmonary heart disease. Specific selection criteria for the DCM VT group included ECG-documented episodes of VT, structural and functional changes as assessed by echocardiography (e.g. a left ventricular ejection fraction of <45% and an end-diastolic dimension (CRA) of the left ventricle more than 6.0 cm). Familial DCM was defined by cases that had at least one additional affected family member with DCM or one case of sudden cardiac death earlier 35 years of age in the pedigree.

Inclusion criteria for the CHD VT group were ECG-documented episodes of VT and established CHD (stable angina II-IV functional class), confirmed by the stress - test and / or coronary angiography, definite fatal and non-fatal myocardial infarction, and other acute coronary syndromes; the latter arbitrary term includes cases when typical history was not accompanied by the occurrence of a Q wave and it corresponds to other common terms such as possible myocardial infarction, minor infarction, intermediate syndrome, acute ischemic attack and non-Q myocardial infarction.
