# Supplementary material for: Patients with coronary heart disease, dilated cardiomyopathy and idiopathic ventricular tachycardia share overlapping patterns of pathogenic variation in cardiac risk genes
Source: PeerJ. 2021 Jan 19;9:e10711. doi: 10.7717/peerj.10711 (PMC7821765; doi:10.7717/peerj.10711)
Supplement: Supplemental Information 3 [file peerj-09-10711-s003.pdf]

SureDesign

HaloPlex Design Report

General Information

User: slave.trajanoski@medunigraz.at
Workgroup: ZMF
Folder: ZMF
File Type: HaloPlex Design Report
Created By: HaloPlex Advanced Design Wizard
Timestamp: 16-Dec-2013

Design Information

Design Name: ZMF96cardio
Design ID: 22408-1387190209
Species: H. sapiens (H. sapiens, hg19, GRCh37, February 2009)
Platform: Illumina
Read Length: 150 bp

Probegroup Summary

Number of Probegroups: 2
Probegroup 1 : CP_241012_opt
Probegroup 2 : ZMF96cardio_1

Target Summary

Target Region Size : 463.767 kbp

Amplicon Summary

Total Amplicons: 19958
Total Target Bases Analyzable: 406.062 kbp
Total Sequenceable Design Size: 1.039 Mbp
Target Coverage: 99.46 %
Recommended Minimum Sequencing per Sample: 207.994 Mbp
Pricing: Illumina Tier 1 (Probe Region Size = 0 - 499 kbp; up to 20K probes)
:

Design Details

Target ID	Regions	Coverage	High Coverage (>= 90%)	Low Coverage (< 90%)
ABCC9	41	100 %	41	0
ACTC1	6	96.97 %	5	1
ACTN2	23	99.82 %	23	0
AKAP9	52	99.72 %	52	0
ANK2	58	99.93 %	58	0
ANKRD1	9	100 %	9	0
ATP2A2	22	100 %	22	0
BAG3	5	100 %	5	0
CACNA1C	53	99.95 %	53	0
CACNA2D1	42	100 %	42	0
CACNB2	20	99.78 %	20	0
CALR3	9	100 %	9	0
CASQ2	11	100 %	11	0
CAV3	2	100 %	2	0
chr3:52485107-52488057	1	100 %	1	0
chr6:7541909-7542329	1	100 %	1	0
chr6:7555939-7556069	1	100 %	1	0
chr6:7558339-7558509	1	100 %	1	0
chr6:7559459-7559649	1	100 %	1	0
chr6:7562869-7563029	1	100 %	1	0
chr6:7563959-7564029	1	100 %	1	0
chr6:7565579-7565769	1	100 %	1	0
chr6:7566609-7566729	1	100 %	1	0
chr6:7567569-7567699	1	100 %	1	0
chr6:7568009-7568149	1	100 %	1	0
chr6:7568659-7568839	1	100 %	1	0
chr6:7569409-7569599	1	100 %	1	0
chr6:7570659-7570809	1	100 %	1	0
chr6:7571609-7571829	1	100 %	1	0
chr6:7572060-7572310	1	100 %	1	0
chr6:7574308-7574499	1	100 %	1	0
chr6:7574879-7575039	1	100 %	1	0

Target ID	Regions	Coverage	High Coverage (>= 90%)	Low Coverage (< 90%)
chr6:7575509-7575732	1	100 %	1	0
chr6:7576507-7576709	1	100 %	1	0
chr6:7577179-7577289	1	100 %	1	0
chr6:7578009-7578129	1	100 %	1	0
chr6:7578679-7578809	1	100 %	1	0
chr6:7579499-7581819	1	100 %	1	0
chr6:7582859-7586129	1	100 %	1	0
CHRM2	1	100 %	1	0
chrx:100645867-100646047	1	100 %	1	0
chrx:100646417-100646567	1	100 %	1	0
chrx:100646727-100646820	1	100 %	1	0
chrx:100650317-100650457	1	100 %	1	0
chrx:100666857-100669138	1	100 %	1	0
chrx:135229548-135229808	1	100 %	1	0
chrx:135252055-135252147	1	100 %	1	0
chrx:135288553-135288758	1	100 %	1	0
chrx:135289161-135289359	1	100 %	1	0
chrx:135289945-135290135	1	100 %	1	0
chrx:135292018-135293528	1	100 %	1	0
chrx:153639866-153640316	1	100 %	1	0
chrx:153640413-153640566	1	100 %	1	0
chrx:153641526-153641606	1	100 %	1	0
chrx:153641806-153641916	1	100 %	1	0
chrx:153647866-153647976	1	100 %	1	0
chrx:153648360-153648446	1	100 %	1	0
chrx:153648536-153648616	1	100 %	1	0
chrx:153648986-153649086	1	100 %	1	0
chrx:153649226-153650076	1	100 %	1	0
CRYAB	3	100 %	3	0
CSRP3	5	99.85 %	5	0
CTF1	3	100 %	3	0
DES	9	100 %	9	0
DMD	88	99.92 %	88	0
DSC2	35	99.82 %	35	0

Target ID	Regions	Coverage	High Coverage (>= 90%)	Low Coverage (< 90%)
DSG2	15	100 %	15	0
DTNA	27	99.68 %	26	1
EMD	6	100 %	6	0
EYA4	21	100 %	21	0
FHL2	8	100 %	8	0
FKTN	12	99.68 %	12	0
GAA	19	100 %	19	0
GJA5	1	100 %	1	0
GPD1L	8	99.18 %	8	0
HCN4	8	100 %	8	0
ILK	12	100 %	12	0
JPH2	6	100 %	6	0
JUP	20	100 %	20	0
KCNA5	1	100 %	1	0
KCND3	7	100 %	7	0
KCNE1	1	100 %	1	0
KCNE2	1	100 %	1	0
KCNE3	1	100 %	1	0
KCNE5	1	100 %	1	0
KCNH2	16	100 %	16	0
KCNJ2	1	99.39 %	1	0
KCNJ5	2	100 %	2	0
KCNJ8	2	99.24 %	2	0
KCNQ1	18	100 %	18	0
LAMA2	67	99.97 %	67	0
LAMA4	44	100 %	44	0
LAMP2	11	99.42 %	11	0
LDB3	16	100 %	16	0
LMNA	17	99.71 %	16	1
MOG1	4	100 %	4	0
MYBPC3	34	100 %	34	0
MYH6	37	97.94 %	35	2
MYH7	38	99 %	37	1
MYL2	7	88.61 %	6	1

Target ID	Regions	Coverage	High Coverage (>= 90%)	Low Coverage (< 90%)
MYL3	6	100 %	6	0
MYLK2	12	100 %	12	0
MYO6	35	99.83 %	35	0
MYOZ2	5	100 %	5	0
MYPN	22	100 %	22	0
NDUFV2	10	100 %	10	0
NEBL	34	100 %	34	0
NEXN	14	100 %	14	0
NKX2-5	5	100 %	5	0
NPPA	3	100 %	3	0
NUP155	35	100 %	35	0
PKP2	14	100 %	14	0
PLN	1	100 %	1	0
PRKAG2	19	99.87 %	19	0
PSEN1	11	99.44 %	11	0
PSEN2	11	100 %	11	0
RBM20	15	99.8 %	15	0
RYR2	111	99.7 %	110	1
SCN1B	5	99.64 %	5	0
SCN3B	5	100 %	5	0
SCN4B	5	100 %	5	0
SCN5A	28	99.58 %	28	0
SGCB	7	100 %	7	0
SGCD	9	100 %	9	0
SNTA1	8	100 %	8	0
TBX20	8	99.8 %	8	0
TBX5	8	100 %	8	0
TCAP	2	100 %	2	0
TGFB3	7	100 %	7	0
TMEM43	13	100 %	13	0
TMPO	10	100 %	10	0
TNNI3	8	100 %	8	0
TNNT2	21	98.44 %	20	1
TPM1	16	99.62 %	16	0

Target ID	Regions	Coverage	High Coverage (>= 90%)	Low Coverage (< 90%)
TTN	364	98.7 %	343	21
TTR	7	100 %	7	0
VCL	22	100 %	22	0
chr2	1	74.5 %	0	1
chr18	1	36.42 %	0	1
chr19	1	58.16 %	0	1
chr2	1	59.14 %	0	1
chr2	1	42.68 %	0	1
chr2	1	61.18 %	0	1
chr2	1	98.82 %	1	0
chr18	1	48.39 %	0	1
chr2	1	99.79 %	1	0
chr4	1	64.73 %	0	1
chr2	1	54.35 %	0	1
chr1	1	94.68 %	1	0
chr2	1	85.67 %	0	1
chr1	1	85.71 %	0	1
chr2	1	34.63 %	0	1
chr2	1	81.47 %	0	1
chr2	1	53.36 %	0	1
chr2	1	36.16 %	0	1
chr2	1	100 %	1	0
chr18	1	65.24 %	0	1
chr2	1	100 %	1	0
chr2	1	67.11 %	0	1
chr18	1	89.4 %	0	1
chr2	1	65.81 %	0	1
chr4	1	98.84 %	1	0
chr2	1	97.79 %	1	0
chr2	1	52.69 %	0	1
chr2	1	74.53 %	0	1
chr12	1	95.38 %	1	0
chr3	1	99.12 %	1	0
chr2	1	89.24 %	0	1

Target ID	Regions	Coverage	High Coverage (>= 90%)	Low Coverage (< 90%)
chr2	1	50.54 %	0	1
chr2	1	100 %	1	0
chr2	1	79.29 %	0	1
chr1	1	79.69 %	0	1
chr2	1	80.43 %	0	1
chr7	1	85.49 %	0	1
chr3	1	95.14 %	1	0
chr2	1	69.37 %	0	1
chr2	1	84.11 %	0	1
chr2	1	81.14 %	0	1
chr2	1	78.37 %	0	1
chr2	1	89.56 %	0	1
chr18	1	60.32 %	0	1
chr1	1	77.38 %	0	1
chr2	1	73.04 %	0	1
chr1	1	58.68 %	0	1
chr7	1	96.11 %	1	0
chr18	1	95.02 %	1	0
chr2	1	98.67 %	1	0
chr10	1	81.14 %	0	1
chr15	1	41.85 %	0	1
chr2	1	96.02 %	1	0
chr2	1	99.26 %	1	0
chr18	1	93.33 %	1	0
chr2	1	55.98 %	0	1
chr17	1	43.75 %	0	1
chr2	1	65.15 %	0	1
chr1	1	93.08 %	1	0
chr2	1	65.51 %	0	1
