# Supplementary material for: Patients with coronary heart disease, dilated cardiomyopathy and idiopathic ventricular tachycardia share overlapping patterns of pathogenic variation in cardiac risk genes
Source: PeerJ. 2021 Jan 19;9:e10711. doi: 10.7717/peerj.10711 (PMC7821765; doi:10.7717/peerj.10711)
Supplement: Supplemental Information 5 [file peerj-09-10711-s005.docx]

**Supplemental file 5.**

**Table S3:**

**Statistical testing of a differential occurrence of HGMD and/or rare variants between the different clinical subgroups. Testing was done including and omitting (excl.) titin variants.**

|  | Groups | N | Median | 25-Percentile | 75- Percentile | p-vaule overall test |
| --- | --- | --- | --- | --- | --- | --- |
| HGMD variants | CHD VT | 23 | 2.00 | 1.00 | 2.00 | 0.870 |
|  | DCM VT | 32 | 2.00 | 1.00 | 2.00 |  |
|  | iVT | 37 | 2.00 | 1.00 | 3.00 |  |
| Rare variants | CHD VT | 23 | 2.00 | 1.00 | 3.00 | 0.719 |
|  | DCM VT | 32 | 2.00 | 1.00 | 3.00 |  |
|  | iVT | 37 | 2.00 | 1.00 | 3.00 |  |
| rare variants incl.TTN | CHD VT | 23 | 3.00 | 2.00 | 4.00 | 1 000 |
|  | DCM VT | 32 | 3.00 | 2.00 | 4.00 |  |
|  | iVT | 37 | 3.00 | 2.00 | 5.00 |  |
| All variants excl.TTN | CHD VT | 23 | 4.00 | 3.00 | 5.00 | 0.708 |
|  | DCM VT | 32 | 4.00 | 2.00 | 5.00 |  |
|  | iVT | 37 | 4.00 | 2.50 | 6.00 |  |
| All variants incl. TTN | CHD VT | 23 | 5.00 | 3.00 | 6.00 | 0.886 |
|  | DCM VT | 32 | 5.00 | 3.25 | 6.75 |  |
|  | iVT | 37 | 5.00 | 2.50 | 7.00 |  |
