# Supplementary material for: Patients with coronary heart disease, dilated cardiomyopathy and idiopathic ventricular tachycardia share overlapping patterns of pathogenic variation in cardiac risk genes
Source: PeerJ. 2021 Jan 19;9:e10711. doi: 10.7717/peerj.10711 (PMC7821765; doi:10.7717/peerj.10711)
Supplement: Supplemental Information 6 [file peerj-09-10711-s006.docx]

**Supplemental file 6.**

**Figure S1a:**

**Prevalence of HGMD variants per clinical subgroup.**

**Figure S1b:**

**Prevalence of rare variants (HGMD variants excluded) per clinical subgroup.**
