# Supplementary figures and images for: Patients with coronary heart disease, dilated cardiomyopathy and idiopathic ventricular tachycardia share overlapping patterns of pathogenic variation in cardiac risk genes
Source: PeerJ. 2021 Jan 19;9:e10711. doi: 10.7717/peerj.10711 (PMC7821765; doi:10.7717/peerj.10711)

**Supplemental file 7.**

**Figure S2:**

**Cumulative relative frequency of class I-III variants per patient.**

**
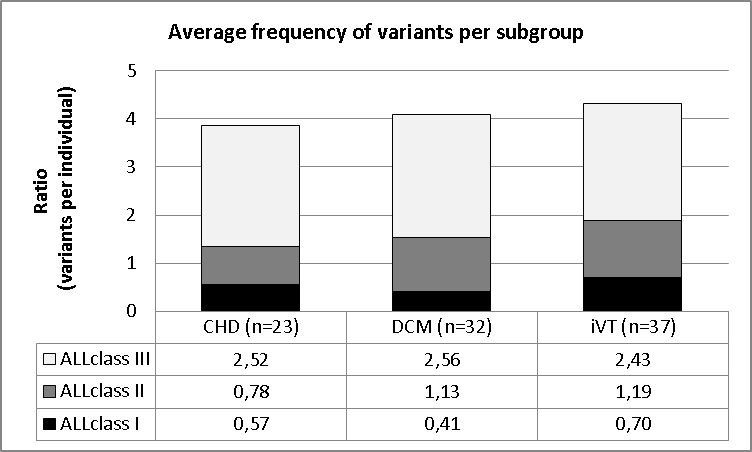
**

Supplement: Supplemental Information 7 [file peerj-09-10711-s007.docx]
