# Supplementary material for: Patients with coronary heart disease, dilated cardiomyopathy and idiopathic ventricular tachycardia share overlapping patterns of pathogenic variation in cardiac risk genes
Source: PeerJ. 2021 Jan 19;9:e10711. doi: 10.7717/peerj.10711 (PMC7821765; doi:10.7717/peerj.10711)
Supplement: Supplemental Information 8 [file peerj-09-10711-s008.docx]

**Supplemental file 8.**

**Table S4:**

**Frequency of HGMD variants per clinical subgroup.**

| HGMD variants | CHD VT | DCM VT | iVT |
| --- | --- | --- | --- |
| no HGMD or addnl. rare SNV | 0 (0%) | 1 (3.1%) | 1 (2.7%) |
| no HGMD | 3 (13.0%) | 5 (15.6%) | 6 (16.2%) |
| 1 HGMD variant | 8 (34.8%) | 4 (12.5%) | 10 (27.0%) |
| 2 HGMD variants | 7 (30.4%) | 14 (43.75%) | 7 (18.9%) |
| 3 HGMD variants | 3 (13.0%) | 5 (15.63%) | 9 (24.3%) |
| ≥ 4 HGMD variants | 2 (8.7%) | 2 (6.25%) | 4 (10.8%) |
