# Supplementary material for: Patients with coronary heart disease, dilated cardiomyopathy and idiopathic ventricular tachycardia share overlapping patterns of pathogenic variation in cardiac risk genes
Source: PeerJ. 2021 Jan 19;9:e10711. doi: 10.7717/peerj.10711 (PMC7821765; doi:10.7717/peerj.10711)
Supplement: Supplemental Information 10 [file peerj-09-10711-s010.docx]

**Supplemental file 10.**

**Table S5:**

**Molecular context of class I-IV variants in the three subgroups.**

| **a) class I** | **CHD VT (n=23)** | **DCM VT (n=32)** | **iVT (n=37)** | **b) class II** | **CHD VT(n=23)** | **DCM VT (n=32)** | **iVT (n=37)** |
| --- | --- | --- | --- | --- | --- | --- | --- |
| cell membrane | 0.00% | 0.00% | 0.00% | cell membrane | 4.30% | 9.40% | 10.80% |
| cytoskeleton | 0.00% | 0.00% | 13.50% | cytoskeleton | 13.00% | 12.50% | 21.60% |
| intercalated disc | 0.00% | 9.40% | 8.10% | intercalated disc | 4.30% | 3.10% | 13.50% |
| ion flux | 13.00% | 6.30% | 18.90% | ion flux | 8.70% | 9.40% | 5.40% |
| metabolism | 8.70% | 0.00% | 5.40% | metabolism | 4.30% | 3.10% | 13.50% |
| nucleus | 13.00% | 6.30% | 8.10% | nucleus | 0.00% | 3.10% | 5.40% |
| sarcomere | 21.70% | 18.80% | 16.20% | sarcomere | 43.50% | 71.90% | 48.60% |
| **c) class III** | **CHD VT (n=23)** | **DCM VT (n=32)** | **iVT (n=37)** | **d) class IV** | **CHD VT (n=23)** | **DCM VT (n=32)** | **iVT (n=37)** |
| cell membrane | 8.70% | 21.90% | 8.10% | cell membrane | 0.00% | 3.10% | 2.70% |
| cytoskeleton | 34.80% | 21.90% | 32.40% | cytoskeleton | 43.50% | 46.90% | 35.10% |
| intercalated disc | 21.70% | 21.90% | 24.30% | intercalated disc | 8.70% | 3.10% | 5.40% |
| ion flux | 21.70% | 40.60% | 21.60% | ion flux | 0.00% | 0.00% | 5.40% |
| metabolism | 47.80% | 18.80% | 29.70% | metabolism | 0.00% | 3.10% | 2.70% |
| nucleus | 34.80% | 31.30% | 43.20% | nucleus | 4.30% | 3.10% | 0.00% |
| sarcomere | 82.60% | 100.00% | 83.80% | sarcomere | 34.80% | 28.10% | 21.60% |
