# Supplementary material for: Patients with coronary heart disease, dilated cardiomyopathy and idiopathic ventricular tachycardia share overlapping patterns of pathogenic variation in cardiac risk genes
Source: PeerJ. 2021 Jan 19;9:e10711. doi: 10.7717/peerj.10711 (PMC7821765; doi:10.7717/peerj.10711)
Supplement: Supplemental Information 11 [file peerj-09-10711-s011.docx]

**Supplemental file 11.**

**Table S6:**

**Gene list representing the relative frequencies of variants and the number of mutations/variants compared to all observed mutations/variants within the subgroup (relative frequency) or related to the subgroup size (frequency per individual).**

| **CHD VT** | | | | **DCM VT** | | | | **iVT** | | | |
| --- | --- | --- | --- | --- | --- | --- | --- | --- | --- | --- | --- |
| Gene ID | var. | Rel. freq. (%) | Freq. per indiv. (%) | Gene ID | var. | Rel. freq. (%) | Freq. per indiv. (%) | Gene ID | var. | Rel. freq. (%) | Freq. per indiv. (%) |
| TTN | 24 | 21.8 | 104.3 | TTN | 38 | 23.8 | 118.8 | TTN | 36 | 19.4 | 97.3 |
| GAA | 10 | 9.1 | 43.5 | LAMA2 | 15 | 9.4 | 46.9 | GAA | 16 | 8.6 | 43.2 |
| LAMA2 | 7 | 6.4 | 30.4 | MYBPC3 | 10 | 6.3 | 31.3 | LAMA2 | 13 | 7.0 | 35.1 |
| MYH6 | 7 | 6.4 | 30.4 | MYH6 | 9 | 5.6 | 28.1 | MYBPC3 | 7 | 3.8 | 18.9 |
| MYBPC3 | 6 | 5.5 | 26.1 | GAA | 7 | 4.4 | 21.9 | DMD | 6 | 3.2 | 16.2 |
| DMD | 5 | 4.5 | 21.7 | ANK2 | 5 | 3.1 | 15.6 | AKAP9 | 6 | 3.2 | 16.2 |
| AKAP9 | 4 | 3.6 | 17.4 | DSG2 | 5 | 3.1 | 15.6 | MYPN | 6 | 3.2 | 16.2 |
| PRKAG2 | 4 | 3.6 | 17.4 | KCNQ1 | 5 | 3.1 | 15.6 | RYR2 | 6 | 3.2 | 16.2 |
| RBM20 | 4 | 3.6 | 17.4 | DMD | 4 | 2.5 | 12.5 | LAMA4 | 5 | 2.7 | 13.5 |
| NPPA | 3 | 2.7 | 13.0 | AKAP9 | 4 | 2.5 | 12.5 | ANK2 | 4 | 2.2 | 10.8 |
| DSP | 3 | 2.7 | 13.0 | RBM20 | 4 | 2.5 | 12.5 | DSG2 | 4 | 2.2 | 10.8 |
| ANK2 | 2 | 1.8 | 8.7 | NPPA | 3 | 1.9 | 9.4 | RBM20 | 4 | 2.2 | 10.8 |
| LAMA4 | 2 | 1.8 | 8.7 | CACNA2D1 | 3 | 1.9 | 9.4 | NPPA | 4 | 2.2 | 10.8 |
| DSG2 | 2 | 1.8 | 8.7 | MYH7 | 3 | 1.9 | 9.4 | DES | 4 | 2.2 | 10.8 |
| MYPN | 2 | 1.8 | 8.7 | LAMA4 | 2 | 1.3 | 6.3 | TBX20 | 4 | 2.2 | 10.8 |
| DSC2 | 2 | 1.8 | 8.7 | MYPN | 2 | 1.3 | 6.3 | MYH6 | 3 | 1.6 | 8.1 |
| JPH2 | 2 | 1.8 | 8.7 | RYR2 | 2 | 1.3 | 6.3 | KCNQ1 | 3 | 1.6 | 8.1 |
| SNTA1 | 2 | 1.8 | 8.7 | DES | 2 | 1.3 | 6.3 | MYH7 | 3 | 1.6 | 8.1 |
| RYR2 | 2 | 1.8 | 8.7 | TNNI3 | 2 | 1.3 | 6.3 | DSP | 3 | 1.6 | 8.1 |
| TMEM43 | 2 | 1.8 | 8.7 | NUP155 | 2 | 1.3 | 6.3 | PKP2 | 3 | 1.6 | 8.1 |
|  |  |  |  | ACTN2 | 2 | 1.3 | 6.3 | NEXN | 3 | 1.6 | 8.1 |
|  |  |  |  | BAG3 | 2 | 1.3 | 6.3 | JPH2 | 2 | 1.1 | 5.4 |
|  |  |  |  | CHRM2 | 2 | 1.3 | 6.3 | CACNA1C | 2 | 1.1 | 5.4 |
|  |  |  |  | GPD1L | 2 | 1.3 | 6.3 | CSRP3 | 2 | 1.1 | 5.4 |
|  |  |  |  |  |  |  |  | CACNB2 | 2 | 1.1 | 5.4 |
|  |  |  |  |  |  |  |  | SNTA1 | 2 | 1.1 | 5.4 |
|  |  |  |  |  |  |  |  | KCNE1 | 2 | 1.1 | 5.4 |
|  |  |  |  |  |  |  |  | DSC3 | 2 | 1.1 | 5.4 |
|  |  |  |  |  |  |  |  | FKTN | 2 | 1.1 | 5.4 |
|  |  |  |  |  |  |  |  | NEBL | 2 | 1.1 | 5.4 |
