# Supplementary material for: Patients with coronary heart disease, dilated cardiomyopathy and idiopathic ventricular tachycardia share overlapping patterns of pathogenic variation in cardiac risk genes
Source: PeerJ. 2021 Jan 19;9:e10711. doi: 10.7717/peerj.10711 (PMC7821765; doi:10.7717/peerj.10711)
Supplement: Supplemental Information 12 — *Statistical testing using Fishers exact t-test suggests a trend for a difference in the frequency of MYH6 and PRKAG2 variants (p-value of 0.053 and 0.054. respectively) between CHD and the other subgroups. [file peerj-09-10711-s012.docx]

**Supplemental file 12.**

**Figure S4:**

**Combined frequency of HGMD mutations and rare variants.**

*Statistical testing using Fishers exact t-test suggests a trend for a difference in the frequency of MYH6 and PRKAG2 variants (p-value of 0.053 and 0.054. respectively) between CHD and the other subgroups.
