# Supplementary material for: Patients with coronary heart disease, dilated cardiomyopathy and idiopathic ventricular tachycardia share overlapping patterns of pathogenic variation in cardiac risk genes
Source: PeerJ. 2021 Jan 19;9:e10711. doi: 10.7717/peerj.10711 (PMC7821765; doi:10.7717/peerj.10711)
Supplement: Supplemental Information 13 [file peerj-09-10711-s013.pdf]

Supplemental file 13.

Figure S5. Sanger sequencing showing gene mutations observed in the present study in patients with VT. Mutation sites are indicated by arrows. Pathogenicity class (ACMG) and #cases - carriers of mutations also shown.

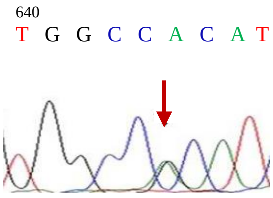

MYBPC3, R160W
c.478C>T, p. Arg160Trp

VUS
iVT #233

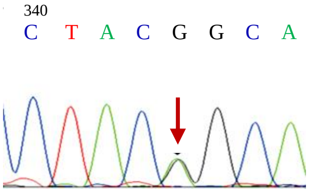

MYBPC3, R326Q
c.977G>A, p.Arg326Gln

Benign
CHM VT #226, #391
DCM VT #210, #278

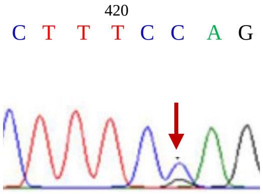

MYBPC3, Q998E
c.2992C>G, p.Gln998Glu

VUS
DCM VT #410, #480
iVT #291

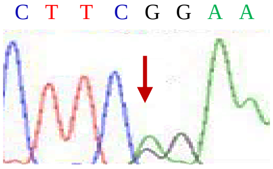

KCNJ2, R218Q
c.653G>A, p.Arg218Gln

Pathogenic
iVT #511

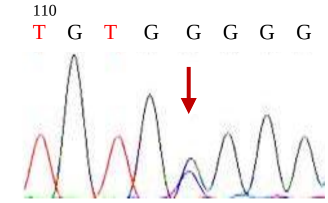

GAA, W746C
c.2238G>C,p.Trp746Cys

Pathogenic
CHD VT #533

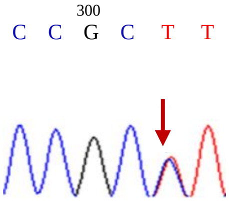

MYH7, F244L
c.730T>C, p.Phe244Leu

Pathogenic
DCM VT #574

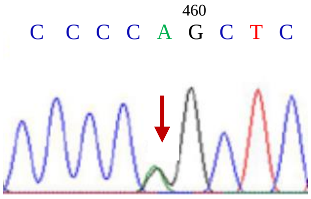

PRKAG2, G100S
c.298G>A, p.Gly100Ser

Benign
CHD VT #344, #436, #412
DCM VT #199
iVT #490

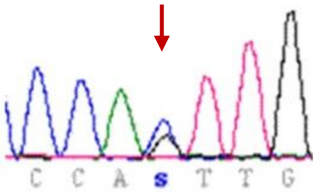

RYR2, V1810L*
c.5428G>C, p.Val1810Leu

Benign
iVT #271

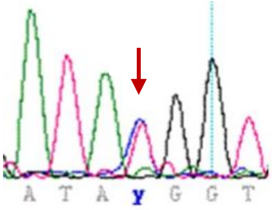

RYR2, T2504M*
c.7511C>T, p.Thr2504Met

Benign
iVT #444

* Akilzhanova A, Guelly C, Nuralinov O, et al. RYR2 sequencing reveals novel missense mutations in a Kazakh idiopathic ventricular tachycardia study cohort. *PLoS One*. 2014;9(6):e101059. Published 2014 Jun 30. doi:10.1371/journal.pone.0101059

Table S7. Sequences of primers used for validation of selected genetic variants.

Gene symbol	Protein/ nucleotide change	Exon		Primer (5' to 3')	PCR fragment (bp)
MYBPC3	R160W c.478C>T, p. Arg160Trp	1	Sense:	GCTCCTCTGCTCCCTACTTCC	708
			Antisense:	ATGGCCATCAGCACACTTCAC	
MYBPC3	R326Q, c.977G>A, p.Arg326Gln	12	Sense:	GGCGGCACAGAGGGGATTG	680
			Antisense:	ACCGGCAGGAGCAAAAGGATG	
MYBPC3	Q998E, c.2992C>G, p.Gln998Glu	27	Sense:	TATGTGACCAGTGGGCAGTTC	1,093
			Antisense:	GGGTCTTGTGACTGCACAAAG	
KCNJ2	R218Q, c.653G>A, p.Arg218Gln	2	Sense:	GGCGAGTGGGCAATCTTCG	470
			Antisense:	CTCAAATCATATAAAGGACTGTC	
GAA	W746C, c.2238G>C,p.Trp746Cys	16	Sense:	CGTTCAACAAGGATGGCTTC	381
			Antisense:	GTGGGTTCTCCAGCTCATTG	
MYH7	F244L, c.730T>C, p.Phe244Leu	8	Sense:	CTTGCTGGTCTCCAGTAGTATTGT	536
			Antisense:	GGCTGAGCCTAGCAGATTCAT	
PRKAG2	G100S, c.298G>A, p.Gly100Ser	3	Sense:	CAGTCCTGTGTGGTCAGAACTTGG	907
			Antisense:	GGACCAGAAGGATTACGCTTTGAT	
RYR2	V1810L, c.5428G>C, p.Val1810Leu	37	Sense:	TGCGTGCTGGCTACTATGAC	410
			Antisense:	CCATTACACAGGAATGCAAAGA	
RYR2	T2504M* c.7511C>T, p.Thr2504Met	49	Sense:	GTAAAACGACGGCCAGTGGCCATTGACACCAAAATTCA	320
			Antisense:	GAAACAGCTATGACCATGACCCATGGCTTACCTGAAAA	
